# Home-based motor imagery intervention improves functional performance following total knee arthroplasty in the short term: a randomized controlled trial

**DOI:** 10.1186/s13018-020-01964-4

**Published:** 2020-10-02

**Authors:** Armin H. Paravlic, Nicola Maffulli, Simon Kovač, Rado Pisot

**Affiliations:** 1 Science and Research Centre Koper, Institute for Kinesiology Research, Koper, Slovenia; 2 Department of Musculoskeletal Disorders, Faculty of Medicine and Surgery, University of Salerno, Salerno, Italy; 3 Centre for Sports and Exercise Medicine, Queen Mary University of London, London, UK; 4 School of Pharmacy and Bioengineering, Keele University School of Medicine, Stoke on Trent, UK; 5 Orthopaedic Hospital Valdoltra, Ankaran, Slovenia; 6 Faculty of Sport, University of Ljubljana, Ljubljana, Slovenia

**Keywords:** Knee osteoarthritis, Total knee replacement, Muscle activation, Cognitive training, Mental simulation, Rehabilitation, Physical function

## Abstract

**Background:**

Motor imagery (MI) is effective in improving motor performance in the healthy asymptomatic adult population. However, its possible effects among older orthopaedic patients are still poorly investigated. Therefore, this study explored whether the addition of motor imagery to routine physical therapy reduces the deterioration of quadriceps muscle strength and voluntary activation (VA) as well as other variables related to motor performance in patients after total knee arthroplasty (TKA).

**Methods:**

Twenty-six patients scheduled for TKA were randomized to either MI practice combined with routine physical therapy group (MIp) or to a control group receiving physical therapy alone (CON). MIp consisted of maximal voluntary isometric contraction (MViC) task: 15 min/day in the hospital, then 5 times/week in their homes for 4 weeks. MViC and VA of quadriceps muscle, knee flexion and extension range of motion, pain level, along with a Timed Up-and-Go Test (TUG) and self-reported measure of physical function (assessed using the Oxford Knee Score questionnaire [OKS]) were evaluated before (PRE) and 1 month after surgery (POST).

**Results:**

Significantly better rehabilitation outcomes were evident on the operated leg for the MIp group compared to CON: at POST, the MIp showed lower strength decrease (*p* = 0.012, *η*^*2*^ *=* 0.237) and unaltered VA, significantly greater than CON (*p* = 0.014, *η*^*2*^ *=* 0.227). There were no significant differences in knee flexion and extension range of motion and pain level (*p* > 0.05). Further, MIp patients performed better in TUG (*p* < 0.001, *η*^*2*^ *=* 0.471) and reported better OKS scores (*p* = 0.005, *η*^*2*^ *=* 0.280). The non-operated leg showed no significant differences in any outcomes at POST (all *p* > 0.05). In addition, multiple linear regression analysis showed that failure of voluntary activation explained 47% of the quadriceps muscle strength loss, with no significant difference in perceived level of pain.

**Conclusion:**

MI practice, when added to physical therapy, improves both objective and subjective measures of patients’ physical function after TKA, and facilitates transfer of MI strength task on functional mobility.

**Trial registration:**

Retrospectively registered on ClinicalTrials.govNCT03684148

## Introduction

Osteoarthritis (OA) is a major cause of disability [1, 2]. Total knee arthroplasty (TKA) successfully relieves pain, corrects deformity, and improves function [3, 4]. However, most patients never reach the functional level of the age-matched OA asymptomatic population even years after surgery [5]. Quadriceps strength is a major determinant of general physical function following TKA [6]. In the early post-surgery period, patients experience loss of more than half of their pre-surgery strength [7, 8], a likely consequence of the alterations of motor control at a central level induced by surgery [7, 9, 10].

Motor imagery (MI)—i.e., the mental representation of a physical action without overt body movement [11]—is effective in improving motor performance [12, 13]. MI exerts beneficial effects on strength [13] and flexibility [14] in healthy adults; it reduces pain [15, 16]; and it contributes to the rehabilitation of Parkinson’s disease and stroke patients [17, 18], though its effects on musculoskeletal patients are equivocal [19]. A recent meta-analysis showed that, when added to routine physical therapy (RPT) in post-injury rehabilitation, MI does not elicit greater benefits on functional mobility, perceived pain, and self-efficiency than RPT alone [19]. However, in older patients following end-stage OA, combining MI with RPT produces positive effects on motor performance following total hip arthroplasty [20] and TKA [21, 22]. An explanation for these apparently contradictory findings may be ascribed to the different MI intervention approaches used, including sample characteristics (e.g., age and individual ability to undertake MI) and/or the primary outcome measures used.

The efficiency of MI relies on the functional equivalence theory suggesting that, when imagining a movement, similar and identical brain areas are activated as if the movement had actually been performed [11, 23]. Therefore, MI might be a suitable addition to routine rehabilitation in the early-post operative period, especially since TKA patients have long-term impaired mobility and difficulty participating in high-intensity exercise training programmes. A few studies investigated the effects of MI practice in TKA patients [15, 21, 22]. For example, by implementing guided imagery (i.e., cognitive training) in post-rehabilitation, Forward et al. [15] showed positive findings for the management of both pain and anxiety, while Jacobson et al. [22] revealed positive effects on gait velocity and reduction of cortisol levels compared to the RPT group [15, 22]. Adding MI to RPT following TKA surgery significantly increased knee extensor muscles’ strength compared to RPT alone [21]. However, none of these studies investigated the possible mechanisms of those positive changes in patients undergoing TKA who undertook MI practice.

Atrophy and a failure of muscle voluntary activation (VA) together explained approximately 85% of the quadriceps maximal voluntary strength loss [7], of which the relative contribution of VA was nearly twice as great as the relative contribution of muscle atrophy to the observed strength decrease at 1 month post-surgery [7]. Indeed, VA is one of the most investigated proxies of central factors related to strength loss following knee surgery. VA is a major factor in the reduction of maximal force output of the muscle, given a patient’s inability to recruit all of the muscle’s motor units, or a failure to attain the maximal discharge rate from the recruited motor units [24]. However, little is known regarding whether MI practice can mitigate the decline in VA following TKA.

In this context, we aimed to explore whether MI, when added to routine physical therapy, may reduce deterioration of maximal voluntary isometric strength (MViC), VA, and other variables related to motor performance, such as functional mobility, the range of motion, and self-reported physical function. We hypothesized a greater deterioration of MViC, VA, and both objective and self-reported measures of physical function in the routine physical therapy group when compared to a MI practice group 1 month post-surgery.

## Methods

### Study design

This was a randomized, controlled, parallel-group intervention trial to evaluate the benefits of adding MI practice to the RPT postoperative rehabilitation programme. Eligible patients were randomly assigned to either an intervention group in which MI practice was combined with RPT (MIp) or to a control group which received RPT alone (CON). Participants were assessed 1 day before surgery and 1 month postoperatively at the Valdoltra Orthopaedic Hospital (Ankaran, Slovenia). Informed consent was obtained from all participants. The study protocol was registered on ClinicalTrials.gov no. NCT03684148.

### Sample size

Isometric knee extension strength of the OA symptomatic leg was defined as the primary outcome variable for the power analysis. The sample size was calculated based on Hopkins recommendations [25] using an online available spreadsheet (http://sportsci.org/resource/stats/index.html). The raw mean difference in change (RDC) was calculated based on our pilot study (RDC = 0.51 Nm/kg; unpublished data). Further, the minimal important difference (MID = 0.19 Nm/kg) was calculated as half of between subjects SD as recommended [26]. As in clinical practice, the drop out of participants is common (ageing process, comorbidities, post-surgery complications); we adjusted the originally calculated sample size by following the formula: N1 = *n*/(1−*d*) [27] where N1 is adjusted sample size, *n* is the sample size required as per the proposed formula (*N* = 10 per group), and *d* is the drop-out rate (*d* = 0.25). This resulted in a sample size of 13 participants in each of the two groups (MIp and control). The recruitment of the patients was continued until the target sample size was achieved (26 in total).

### Randomization and blinding

For allocation of the participants, a computer-generated list of random numbers (1 or 2) was produced using Excel 2016 (Microsoft, Redmond, WA, USA). Group assignment occurred after inclusion criteria were met and prior to the preoperative testing session. Checking for eligibility and group allocation was performed by dedicated medical staff who was not otherwise involved in the clinical management of the patients. Testers were not blinded to group assignment because resources did not permit the hiring of separate personnel for testing and MI practice treatments. The surgeons and the remaining medical staff, including physical therapists, nurses, and administration, were unaware of the group allocation of the patients.

### Participants characteristics

**Fig. 1:**
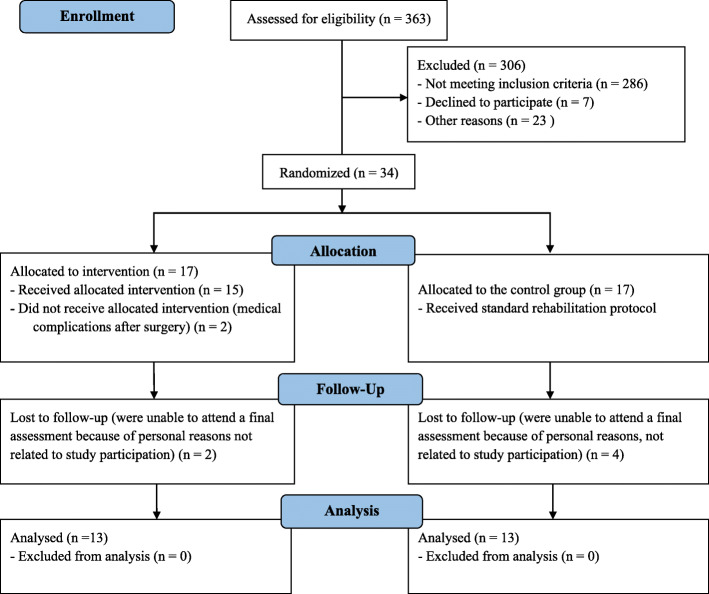
Flow chart of participant enrolment, randomised group allocation, and final analysis

Patients who were scheduled for a primary unilateral TKA by three orthopaedic surgeons at the Valdoltra Orthopaedic Hospital (Ankaran, Slovenia) were consecutively recruited between August 2017 and April 2018. All patients underwent a tricompartmental, cemented TKA using a medial parapatellar approach. Patients were included if they were aged 50 to 85 years, suffered from end-stage OA of the knee, were scheduled for unilateral TKA, were free from OA or other musculoskeletal problems on the contralateral lower limb, and had not been involved in pre-operative motor imagery treatment. A total of 26 participants successfully completed both (PRE and POST) measurements (for baseline characteristics, see Table 1). The enrolment, randomization, and final analysis procedures are shown in the CONSORT flow diagram (Fig. 1). Exclusion criteria were participants who were undergoing a revision TKA; those with a body mass index (BMI) greater than 40 kg/m^2^; participants who were receiving a bilateral TKA; those with uncontrolled hypertension, diabetes mellitus, a history of any neurological disorder, multiple sclerosis, Parkinson’s disease; patients with rheumatoid arthritis or active cancer; previous history of deep vein thrombosis; contralateral knee OA (as defined by pain greater than 4/10 with activity). All procedures were carried out in accordance with the ethical standards of the 1964 Declaration of Helsinki and were approved by the Ethics Committee of Valdoltra Orthopaedic Hospital (approval no. 16/2016).

**Table 1:** Participants' characteristics at baseline

		MIp group (*n* = 13)	Control group (*n* = 13)	Drop-outs (*n* = 8)	ANOVA (*p*)
**Demographic characteristics**					
Age (years)		61.69 ± 5.19	58.85 ± 5.24	63.88 ± 4.73	0.096
Sex (men/women)		7/6	7/6	5/3	
BMI (kg/m^2^)		30.54 ± 4.03	30.15 ± 1.8	29.12 ± 3.56	0.615
Total knee arthroplasty, (right side, n)		8/13	7/13	5/8	
Days of hospital stay		8.77 ± 1.74	8.23 ± 1.83	8.50 ± 1.6	0.737
**Physical function**					
MViC extension (Nm/kg)	Operated leg	1.37 ± 0.35	1.51 ± 0.38	1.49 ± 0.47	0.657
	Non-operated leg	1.77 ± 0.32	1.63 ± 0.36	1.98 ± 0.56	0.170
Voluntary muscle activation (%)	Operated leg	80.08 ± 13.28	80.86 ± 14.13	87.08 ± 7.36	0.433
	Non-operated leg	85.83 ± 9.48	82.44 ± 9.65	85.90 ± 6.79	0.567
Knee flexion (degrees)	Operated leg	87.69 ± 9.66	86.23 ± 18.08	96.0 ± 18.24	0.352
	Non-operated leg	104.23 ± 8.16	105.54 ± 13.08	105.75 ± 12.51	0.940
Knee extension (degrees)	Operated leg	4.31 ± 3.25	3.08 ± 2.63	3.50 ± 2.67	0.554
	Non-operated leg	2.54 ± 1.81	1.38 ± 1.56	3.0 ± 1.77	0.089
VAS score (points)	Operated leg	53.85 ± 12.1	54.62 ± 14.21	52.5 ± 18.9	0.950
	Non-operated leg	8.46 ± 8.99	14.62 ± 13.61	9.38 ± 7.76	0.321
Hand grip strength, dominant arm (kg)		39.77 ± 9.61	36.54 ± 11.93	43.12 ± 10.41	0.398
TUG (s)		7.48 ± 1.52	7.57 ± 1.55	7.78 ± 1.75	0.914
OKS score (points)		21.92 ± 5.25	22.38 ± 6.23	26.0 ± 9.04	0.365

*BMI* Body mass index, *MIp* Motor imagery practice group, *CON* Control group, *n* number, *TUG* Timed Up-and-Go Test, *OKS* Oxford Knee Score questionnaire; One-Way ANOVA did not reveal any significant difference between groups at baseline, *p* level of significance

### Interventional programmes

The RPT and MIp intervention are described using the Template for Intervention Description and Replication (TIDieR) in Table 2 [28].

**Table 2:** Study intervention description based on the TIDieR checklist

Item		Experimental group	Control group
1	Brief name	Motor imagery practice (MIp) + routine physical therapy (RPT)	RPT (usual care) alone
2	Why	Both interventions were compared directly in OA patients submitted to TKA with following reasons: 1) TKA patients are unable to undertake conventional strength training in the early period following surgery, whereas quadriceps strength is a major determinant of general physical function following TKA. 2) MIp does not elicit pain or any side effects during practice, nor does it require any additional fees and special condition, except a quiet space where the trainee/patient can relax and train. 3) It is assumed that strength decrease following TKA is largely influenced by plasticity in neural drive (central level), rather than peripheral level. Thus, including MIp in addition to RPT may have a positive effect on maximal voluntary activation level (a proxy of the central neural drive). 4) When added to RPT, MIp might have favourable effects on both task-specific (near transfer) and general physical function (far transfer), which remains unclear in TKA population. 5) If so, MIp might be a suitable adjunct tool to RPT intended to improve rehabilitation of TKA patients, without additional costs for patients and the health care system.	
3	What: materials	No restriction was placed on materials used (for example: bed, chair, pillow, crutches, steppers, stairs), while the use of additional mechanical (for example: continuous passive motion—only allowed during hospitalization) or electrical therapy devices (for example: neuromuscular stimulation) was avoided.	
		Hospitalization: patients had one-to-one therapy (MIp) in sitting position (based on the current patient’s physical state). The therapist guided the patient throughout the practice protocol. After hospital discharge (at home): MIp practice was delivered by audio mp3 file.	Hospitalization: the patients engaged in conversation with the therapist about their health status, rehabilitation progression based on predefined goals. After hospital discharge (at home): none in particular, the patients were called by phone (3× per week, on consecutive days) and asked about their subjective health status, treatment adherence and rehabilitation progress.
4	What: procedures	During hospitalization, all the enrolled patients underwent the same functional exercise-based rehabilitation programme aimed to improve knee range of motion, increase knee and hip muscle strength, stretch the posterior and anterior aspect of the thigh muscles, prevent thrombosis, and help acquire the most important functional strategies for activities of daily living. First, the subjects received one daily continuous passive motion (CPM) session (Kinetec Performa), beginning on the second day after TKA (after recovery unit) until discharge (4 to 8 days). The CPM session lasted 45 min, including a 5-min warm-up period. Further, the exercise programme consisted of 60 min of one-to-one therapy: 5–10 min warm-up and cool-down periods including passive and active stretching of lower limb muscle groups; knee flexion (heel sledge in bed); plantar flexion of ankles (supine); hip abduction and adduction (supine); supine straight leg raises (for the operated leg—the patients used the help of contralateral leg); walking with aids, sit-to-stand from chairs of various heights (exercises adopted based on injured knee flexion and pain level); standing calf raises; standing hip flexion and extension; walking up and down the stairs (using crutches and/or handrail), arm raises, and shoulder range of motion.	
		MIp additionally performed a mental simulation of maximal isometric contraction only. Patients were instructed to sit on a chair and to imagine the operated leg flexed at 60° at the knee joint while listening to the therapist or to an audio tape with detailed practice instructions.	
5	Who provides	During the hospitalization period, the exercise programme was provided by experienced physical therapist blinded for patients’ intervention allocation. Home-based intervention was conducted by patients themselves.	
6	How	Both interventions were conducted individually in one-to-one sessions (during the hospitalization period), whereas following hospital discharge the patients trained alone.	
7	Where	Both interventions took place in the hospital (orthopaedic ward programme) and at patients’ homes.	
8	When and how much	The programme began on the second post-surgery day and lasted 4 weeks in total. The first part of the programme was performed during the hospitalization period (6 days on average), whereas after the hospital discharge the patients continued with the allocated intervention at their homes. All patients, regardless of their allocation to groups, performed an RPT programme 5 times a week, 2 times per day (lasting 45 to 60 min per session). Each exercise was planned in a progressive manner, meaning: -Strength exercises: starting with two sets and 10 repetitions per week—then adding 2 repetitions in the second and 3 and 5 more repetitions in the third week and fourth week, respectively; -Stretching exercises: starting with 3 repetitions and 15-s holds per week—then adding 5 more seconds each week; -Walking exercise: trying to walk for 10 min on level ground—then adding 5 min every week.	
		MIp was planned in a progressive manner. It was performed in two sets of 25 repetitions with 2 min of inter-set rest period, for two weeks, with 10 repetitions added in weeks 3 and 4, respectively. Each MViC repetition was sustained for 5 s and followed by a 5-s inter-repetition rest period. Additionally, after every fifth contraction, participants had 20 s of rest to minimize mental fatigue. Following 5 days of MI practice, the participants were advised to take a break from MI for two consecutive days.	
9	Tailoring	The exercise programme content was tailored to each patient’s preferences based on their self-perceived level of pain and current function (mainly knee flexion movement).	
10	Modifications	No modification occurred during the study.	
11	How well	Regardless of group assignment, participants were called by principal investigator on a weekly basis and monitored for adherence to the prescribed treatment for both RPT and MIp sessions.	
12		The adherence to the prescribed MI post-rehabilitation was as high as 98%.	The adherence to RPT was high, 98% and 96% for MIp and CON group, respectively.

### Hospitalization period

During hospitalization, all the enrolled patients underwent the same functional exercise-based rehabilitation programme for improving knee range of motion, increasing knee and hip muscle strength, stretching the posterior and anterior aspects of the thigh muscles, preventing thrombosis, and acquiring the most important functional strategies for activities of daily living.

### Home-based intervention

After hospital discharge, both groups were supplied with a physical exercise programme booklet (Supplementary 1) of the same exercises as those performed while in hospital and instructed to perform them at home. Each participant was contacted by phone every other day and monitored for adherence to the prescribed exercise programme.

As the reported placebo effect in psychological outcomes of exercise training is small (ES = 0.20) [29] to control the latter and the additional socio-psychological influence of the MI practice instructor on the MI outcomes, we ensured the same conditions for the control group, and spent the same amount of time with each patient (approximately 15 min per day in the form of verbal communication on site/in hospital or by telephone call/after discharge) regardless of patients’ randomization allocation.

### Experimental group

**Fig. 2:**
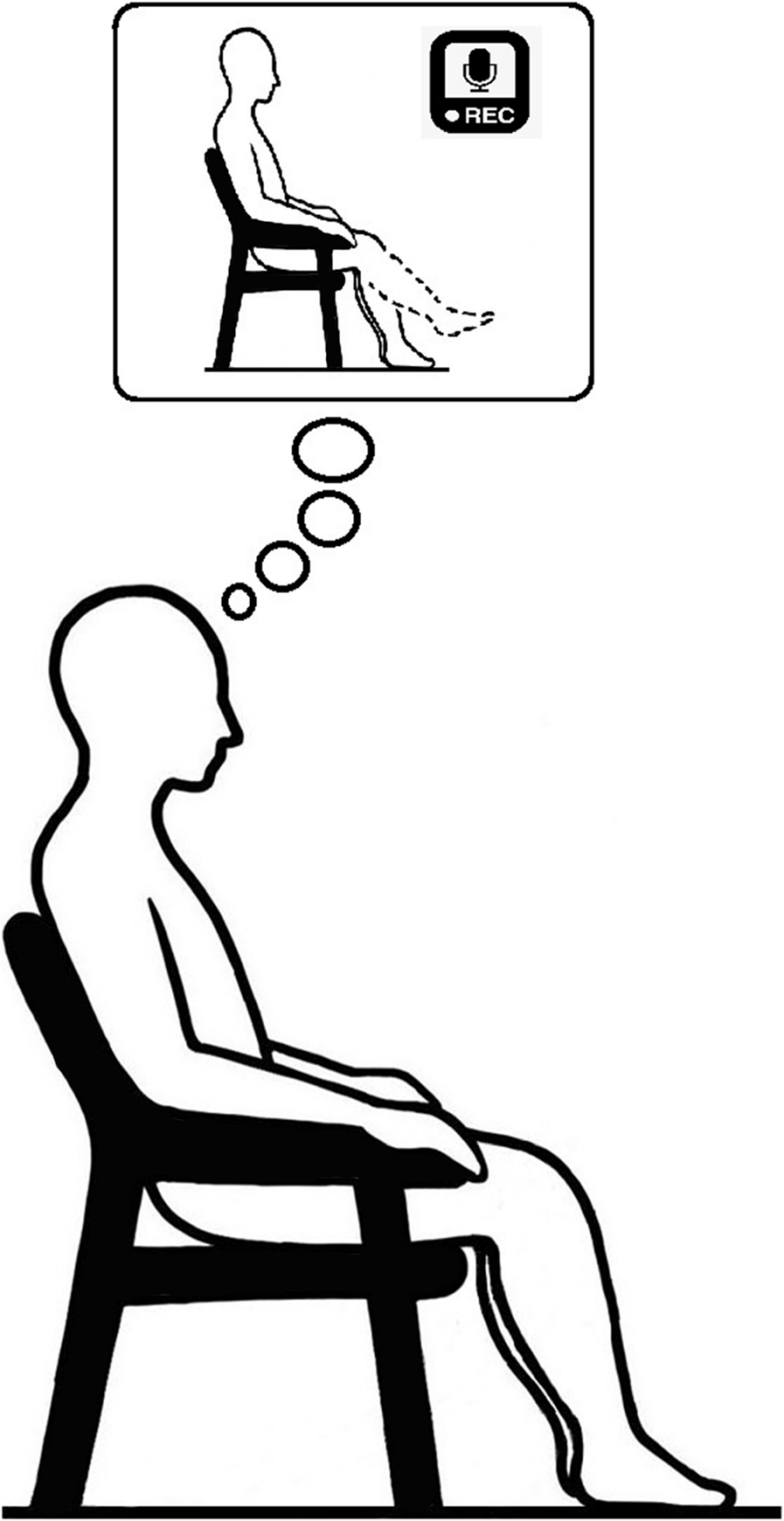
Motor imagery training setup

Patients included in the MIp received an additional intervention based on motor imagery beginning immediately after the TKA procedure. Specifically, they were advised to imagine MViC, the same ones as during measurement settings. MViC imagery practice was planned in a progressive manner (see Table 2). Briefly, the patients performed two sets of 25 repetitions with 2 min of inter-set rest periods, for a duration of 2 weeks. To comply with the basic principles of strength training i.e., step-by-step overload and progression, 10 additional trials were added during weeks 3 and 4, respectively. Each MViC repetition was sustained for 5 s, followed by a 5-s inter-repetition rest period. Additionally, after every fifth contraction, the participants had 20 s of rest. Following 5 days of MI practice, the participants were advised to take a break from MI for two consecutive days. The MI training setup is illustrated in Fig. 2.

#### Control group

Patients from the control group underwent the same post-surgery rehabilitation programme described above but did not engage in MI practice.

### Assessments

All measurements were carried out in a separate and quiet room to avoid any external disturbances from the hospital environment. All tests were performed twice, 1 day prior to surgery (PRE) and 1 month post-surgery (POST).

#### Primary outcomes

##### Knee extensor muscles testing

Subjects were seated upright and firmly strapped down in a custom-built dynamometer (S2P Ltd., Bled, Slovenia) with their hips and knee flexed at 110° and at 60° joint angle. A steel cuff was strapped around the lower leg ~ 2 cm above the medial malleoli and connected to a strain-gauge load cell (Z6FC3–200kg, HBM, Darmstadt, Germany), while arms were crossed at chest level. Individual dynamometer settings were recorded to ensure identical subject positioning at all test sessions (PRE and POST).

##### Maximal voluntary isometric strength and voluntary activation of knee extensor muscles

**Fig. 3:**
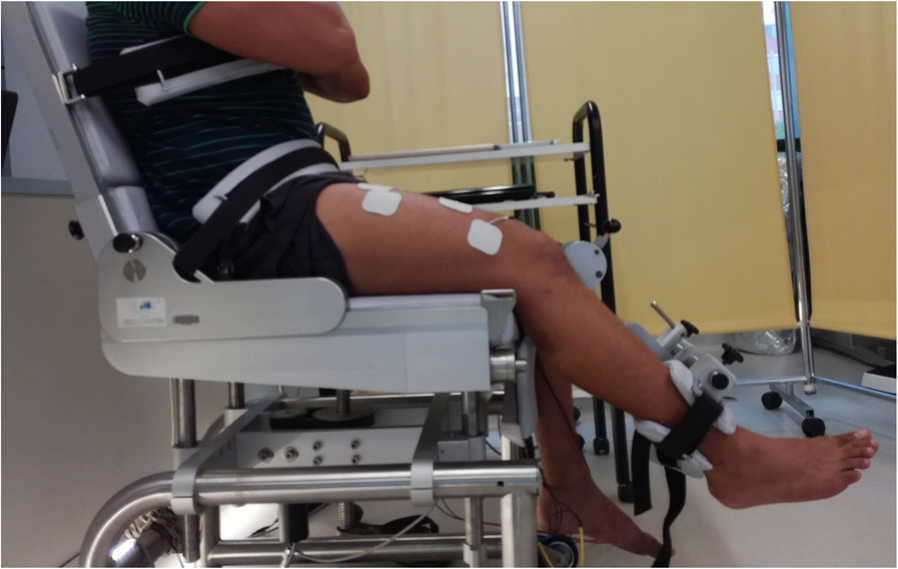
Experimental setup for the measurement of isometric strength and voluntary activation of the quadriceps femoris muscle

Measurement of MViC of the quadriceps muscle was assessed by using the superimposed double-twitch technique [30]. Each subject performed two submaximal contractions (approximately 50 and 75% of self-perceived maximal effort) and one maximal voluntary contraction lasting 2 to 3 s each to warm up the muscle and gain familiarity with the testing procedure. After 3 min of rest, the subjects were instructed to perform maximal isometric knee extensions, to contract as fast and forcefully as possible and to maintain maximal force exertion until a plateau in force production was reached. Approximately 2 s into the contraction and 3 s after, a “supramaximal” double twitch stimulation in the resting muscle was manually delivered by the stimulator. The measurements were conducted using a constant-current electrical stimulator (National Instruments) for stimulating the quadriceps femoris throughout the stimulation of three superficial muscles (rectus femoris, vastus medialis, and vastus lateralis) by self-adhesive 5 × 5 and 2-mm-thick electrodes (dual-stick) located on the proximal and distal portions of individual muscles. Two biphasic, symmetric, and squared electrical impulses (double twitch) of 0.3 ms duration of supramaximal intensity were used at an interval of 10 ms. During the actual measurement, the knee was additionally fixed over the medial and lateral epicondyles of the femur and the hip (Fig. 3). Strong verbal encouragement and visual feedback of the dynamometer force response were provided during all trials. All MViC trials (up to three per subject) were separated by 2 min of rest. Trials with a visible initial countermovement (i.e., drop of the force above 0 value) were discarded. The trial with the highest superimposed peak force was selected as representative of MViC. Voluntary muscle activation was calculated according to the formula proposed by Strojnik and Komi [31]:

$$ \mathrm{VA}\kern0.5em \left(\%\right)\kern0.5em =\kern0.5em 1\hbox{-} \left(\frac{\mathrm{D}\ast \left(\frac{{}^{\mathrm{F}}\mathrm{S}\mathrm{tim}}{{}^{\mathrm{F}}\mathrm{M}\mathrm{ViC}}\right)}{{}^FS timRest}\right)\ast 100 $$

where *D* is the difference between force at stimulation (*F*_Stim_) and the force peak evoked by stimulation onto the MViC, *F*_MViC_ is force at MVC, and *F*_StimRest_ is force achieved due to stimulation in the potentiated resting muscle.

MViC was normalized to body mass, as maximal strength is a variable dependent on body size [32].

#### Secondary outcomes

##### Timed Up-and-Go Test

The TUG test was administered to quantify the functional mobility of patients [33]. The patients were asked to rise from the chair (seat height 46 cm), walk around an obstacle that was 3 m away, turn around, and return to take a seat as quickly and safely as possible. Patients were instructed to walk as quickly as they could while feeling both safe and comfortable. A stopwatch was used to measure the time to complete the TUG within the nearest one-hundredth of a second. The TUG measures mobility in older adults with excellent test-retest reliability (intraclass correlation coefficient [ICC] = 0.97) [34]. A practice trial was given, and then two timed trials were recorded and averaged [20].

##### Range of motion

Knee ROM was measured using a standard long-arm goniometer, as previously reported [35]. The axis of the goniometer was aligned with the centre of the lateral epicondyle of the femur. The distal arm of the goniometer was aligned with the lateral malleolus and the proximal arm was aligned with the greater trochanter of the femur. To determine knee flexion ROM, patients were supine and asked to actively slide the heel towards the buttocks. The angle of maximal active knee flexion was measured. Knee extension ROM was also assessed while supine, with the patient’s heel propped off the treatment table on a 15-cm-thick hard foam pad. Subjects were asked to activate the quadriceps, extending the knee. The angle of the maximal extension was recorded. Positive values were used to indicate a position of flexion at maximal knee extension, and negative numbers were used to represent hyperextension. Knee ROM measured in patients with knee OA has adequate reliability with a coefficient of 0.96 for flexion and 0.81 for extension [36].

##### Knee pain

A numeric rating scale was used to quantify knee pain during rest as well as during burst-superimposition testing. Subjects were asked to verbally rate the pain in and around the knee during the burst superimposition test on a scale from 0 to 100, with 0 representing no pain and 100 representing the worst pain imaginable [37].

##### Maximal grip strength

Maximal grip strength was measured bilaterally with a portable Jamar Hydraulic Hand Dynamometer (Sammons Preston, Rolyan, Bolingbrook, IL, USA). In accordance with American Society of Hand Therapy recommendations, subjects were seated with their shoulders in 0° abduction and neutral rotation, their elbows in 90° of flexion, and their forearms in neutral pronation/supination. The average of three and two maximal repetitions was used for further analysis.

##### Self-reported physical function

The Oxford Knee Score (OKS) questionnaire was used to evaluate the self-reported physical function of patients with different knee pathologies and following TKA surgery [38]. Each of the 12 questions on the OKS is scored in the same way with the score decreasing as the reported symptoms increase (i.e., become worse), that is the highest result of 60 points represents no symptoms at all.

### Data analysis

The data were analysed with IBM SPSS Statistics 24.0 software for Windows (SPSS Inc., Chicago, IL, USA). Normality of distribution was confirmed by visual inspection and using the Shapiro-Wilk test, while the homogeneity of variances was tested using Levene’s test for all dependent variables. To minimise the results of interpretation bias due to patients lost to follow-up, three-fold analysis including the initial comparison of three groups (MIp vs CON vs Drop-outs), intention-to-treat and per-protocol analysis were utilised as recommended [31, 32]. Therefore, a one-way analysis of variance (ANOVA) was used to examine whether participants who completed the study protocol differed from those who did not (i.e., dropouts *N* = 8). Further, mixed-effect models were used to examine the primary and secondary hypotheses using the intention-to-treat approach, allowing us to incorporate all patients who were originally randomly assigned to their groups (including data from patients with missing values). The maximum likelihood estimation method for missing values was used in the mixed effects models. Missing data (*N* = O from baseline; *N* = 8 from final measurements) was not imputed. In all models, the fixed factor was group assignment (MIp vs. CON), with two-time points (PRE vs. POST). Interactions were tested by a two-way analysis of variance (ANOVA) where the group (MIp and CON) was used as the between-subject factor and time (PRE and POST measurements) as the within-subject factor. In case of significance,post hoc comparisons were performed. In addition, we performed multiple linear regression analysis to investigate the contribution of changes in voluntary activation and pain to the changes in quadriceps strength. Further, magnitude-based inferences were determined by quantifying the chances that true differences in comparisons were greater, similar to, or smaller than the smallest significant difference and interpreted qualitatively as *most unlikely* = < 0.5%; *very unlikely* = 0.5–5%; *unlikely* = 5–25%; *possibly* = 25–75%; *likely* = 75–95%; *very likely* = 95–99.5%; *most likely* = > 99.5% [39]. Statistical significance was set at the level of *p* < 0.05 [39].

## Results

### Study population

Three hundred sixty-three patients scheduled for TKA at Valdoltra Orthopaedic Hospital were assessed for eligibility. Three hundred six patients were excluded: 286 did not satisfy the inclusion criteria, 7 patients declined to participate, and 23 patients were excluded for various other reasons (pre-operative comorbidities; inability to attend for post-operative measurements). Therefore, 26 patients (14 males and 12 females) were enrolled in the study (details depicted in Fig. 1). There were no differences between groups in baseline demographic data (*p* > 0.05, Table 1). No patient reported adverse events resulting from participation in the study. The adherence to prescribed post-rehabilitation was high (98%). Functional outcome data did not violate normality of distribution as assessed by the Shapiro-Wilk test (all *p* ≥ 0.071). Furthermore, the independent-samples *t* test showed no significant differences between the intervention and control groups in any outcome parameter at baseline.

Overall findings regarding performance and self-reported measures of MI practice and control groups before and 1 month after surgery are presented in Tables 3 and 4.

**Table 3:** Mixed effects models for physical performance and self-reported measures (intention to treat analysis)

Variable						LMEM time effect		Time^a^group effect	
			PRE (*N* = 17)	POST (*N* = 13)	**Δ (%)**	Parameter estimate (SE)	*t* value (*p* value)	**Parameter estimate (SE)**	*t* value (*p* value)
MViC extension (Nm/kg)	Operated leg	MIp	1.35 ± 0.41	0.80 ± 0.34	− 42.3 62.5	0.98 (0.08)	11.801 (**< 0.001**)	− 0.42 (0.12)	− 3.541 (**0.001**)
		CON	1.55 ± 0.35	0.55 ± 0.25	− 62.5				
	Non-operated leg	MIp	1.80 ± 0.39	1.77 ± 0.29	1.2	− 0.008 (0.042)	− 0.205 (0.839)	0.008 (0.060)	0.129 (0.899)
		CON	1.74 ± 0.44	1.66 ± 0.35	2.2				
Voluntary muscle activation (%)	Operated leg	MIp	81.63 ± 12.48	84.84 ± 9.75	8.3	17.91 (5.10)	3.513 (**0.001**)	− 21.26 (7.21)	− 2.949 (**0.006**)
		CON	82.42 ± 12.91	64.36 ± 20.85	− 16.7				
	Non-operated leg	MIp	85.26 ± 9.23	81.42 ± 12.44	− 3.2	− 0.22 (3.98)	− 0.056 (0.956)	4.05 (5.63)	0.720 (0.477)
		CON	83.84 ± 8.78	84.08 ± 12.78	2.5				
Knee flexion (degrees)	Operated leg	MIp	91.41 ± 12.81	82.31 ± 9.75	− 5.6	14.38 (4.50)	3.197 (**0.03**)	− 5.79 (6.36)	− 0.910 (0.370)
		CON	86.76 ± 17.81	72.31 ± 14.28	− 13.2				
	Non-operated leg	MIp	106.88 ± 9.64	106.54 ± 9.93	2.4	− 2.51 (2.91)	− 0.862 (0.396)	1.71 (4.12)	0.416 (0.681)
		CON	103.29 ± 12.16	106.77 ± 11.10	2.1				
Knee extension (degrees)	Operated leg	MIp	4.12 ± 3.18	7.15 ± 3.74	45.9	− 3.20 (1.32)	− 2.42 (**0.022**)	0.15 (1.87)	0.081 (0.936)
		CON	3.18 ± 2.51	6.38 ± 4.56	101.3				
	Non-operated leg	MIp	2.76 ± 1.79	2.15 ± 1.86	0.6	− 0.21 (0.75)	− 0.28 (0.784)	0.88 (1.07)	0.823 (0.417)
		CON	1.65 ± 1.66	1.92 ± 2.36	8.3				
VAS score (points)	Operated leg	MIp	53.55 ± 12.84	31.54 ± 12.14	− 39.8	16.93 (4.32)	3.92 (**0.001**)	5.13 (6.11)	0.840 (0.408)
		CON	54.12 ± 15.93	37.31 ± 14.67	− 27.3				
	Non-operated leg	MIp	8.24 ± 8.83	1.54 ± 3.76	− 83.3	6.94 (2.26)	3.070 (**0.004**)	− 0.12 (3.20)	− 0.039 (0.970)
		CON	13.82 ± 12.19	7.31 ± 13.01	− 56.0				
Hand grip strength, dominant arm (kg)		MIp	36.53 ± 12.92	38.85 ± 7.41	− 0.8	0.45 (1.15)	0.391 (0.699)	− 0.13 (1.63)	− 0.079 (0.937)
		CON	40.24 ± 12.42	36.77 ± 11.41	2.7				
TUG (s)		MIp	7.72 ± 1.61	7.55 ± 1.22	4.0	− 4.02 (0.58)^a^	− 6.960 (**< 0.001**)	4.04 (0.82)	4.952 (**< 0.001**)
		CON	7.45 ± 1.51	11.54 ± 3.01	54.4				
OKS score (points)		MIp	23.12 ± 5.99	27.08 ± 5.17	28.3	− 3.22 (1.82)^a^	− 1.767 (0.087)	5.27 (2.49)	2.116 (**0.043**)
		CON	23.0 ± 7.44	20.08 ± 4.27	− 5.2				

*BMI* Body mass index, *MBI* Magnitude-based inference, *RDC* Raw mean difference in change, *Δ* (%)–percent changes between initial and final measurement, *SE* Standard error, *LMEM* Linear mixed effects models *p* level of significance

^a^Negative direction of effect means better result on test;

*Note*: Data are presented as mean ± SD; *Bold* values represent a significant effect

**Table 4:** Performance and self-reported measures of motor imagery practice (Mip) and control (CON) groups before and 1 month after surgery (per protocol analysis)

Variable			PRE (*N* = 13)	POST (*N* = 13)	RDC (95% CI)	MBI
MViC extension (Nm/kg)	Operated leg	MIp	1.37 ± 0.35	0.80 ± 0.34	0.39 (0.03, 0.75)	Likely beneficial
		CON	1.51 ± 0.38	0.55 ± 0.25		
	Non-operated leg	MIp	1.77 ± 0.32	1.77 ± 0.29	− 0.03 (− 0.39, 0.33)	Possibly negative
		CON	1.63 ± 0.36	1.66 ± 0.35		
Voluntary muscle activation (%)	Operated leg	MIp	80.08 ± 13.28	84.84 ± 9.75	21.26 (4.90, 37.62)	Very likely beneficial
		CON	80.86 ± 14.13	64.36 ± 20.85		
	Non-operated leg	MIp	85.83 ± 9.48	81.42 ± 12.44	− 6.05 (− 18.22, 6.12)	Unlikely negative
		CON	82.44 ± 9.65	84.08 ± 12.78		
Knee flexion (degrees)	Operated leg	MIp	87.69 ± 9.66	82.31 ± 9.75	8.54 (− 6.04, 23.12)	Likely beneficial
		CON	86.23 ± 18.08	72.31 ± 14.28		
	Non-operated leg	MIp	104.23 ± 8.16	106.54 ± 9.93	1.08 (− 10.57, 12.73)	Possibly beneficial
		CON	105.54 ± 13.08	106.77 ± 11.10		
Knee extension (degrees)	Operated leg	MIp	4.31 ± 3.25	7.15 ± 3.74	− 0.46 (− 4.39, 3.47)	Possibly negative
		CON	3.08 ± 2.63	6.38 ± 4.56		
	Non-operated leg	MIp	2.54 ± 1.81	2.15 ± 1.86	− 0.93 (− 3.02, 1.16)	Unlikely negative
		CON	1.38 ± 1.56	1.92 ± 2.36		
VAS score (points)	Operated leg	MIp	53.85 ± 12.1	31.54 ± 12.14	− 5.00 (− 19.49, 9.49)	Unlikely negative
		CON	54.62 ± 14.21	37.31 ± 14.67		
	Non-operated leg	MIp	8.46 ± 8.99	1.54 ± 3.76	− 0.39 (− 11.91, 11.13)	Possibly negative
		CON	14.62 ± 13.61	7.31 ± 13.01		
Hand grip strength, dominant arm (kg)		MIp	39.77 ± 9.61	38.85 ± 7.41	− 1.15 (− 12.29, 9.99)	Possibly negative
		CON	36.54 ± 11.93	36.77 ± 11.41		
TUG (s)		MIp	7.48 ± 1.52	7.55 ± 1.22	− 3.90 (− 6.02, − 1.78)*	Most likely beneficial
		CON	7.57 ± 1.55	11.54 ± 3.01		
OKS score (points)		MIp	21.92 ± 5.25	27.08 ± 5.17	7.46 (1.72, 13.20)	Very likely beneficial
		CON	22.38 ± 6.23	20.08 ± 4.27		

*BMI* Body mass index, *MBI* Magnitude-based inference, *RDC* Raw mean difference in change

^*^Negative direction of effect means better result on test

### Knee extensor muscles isometric strength and voluntary activation

#### Operated leg

**Fig. 4:**
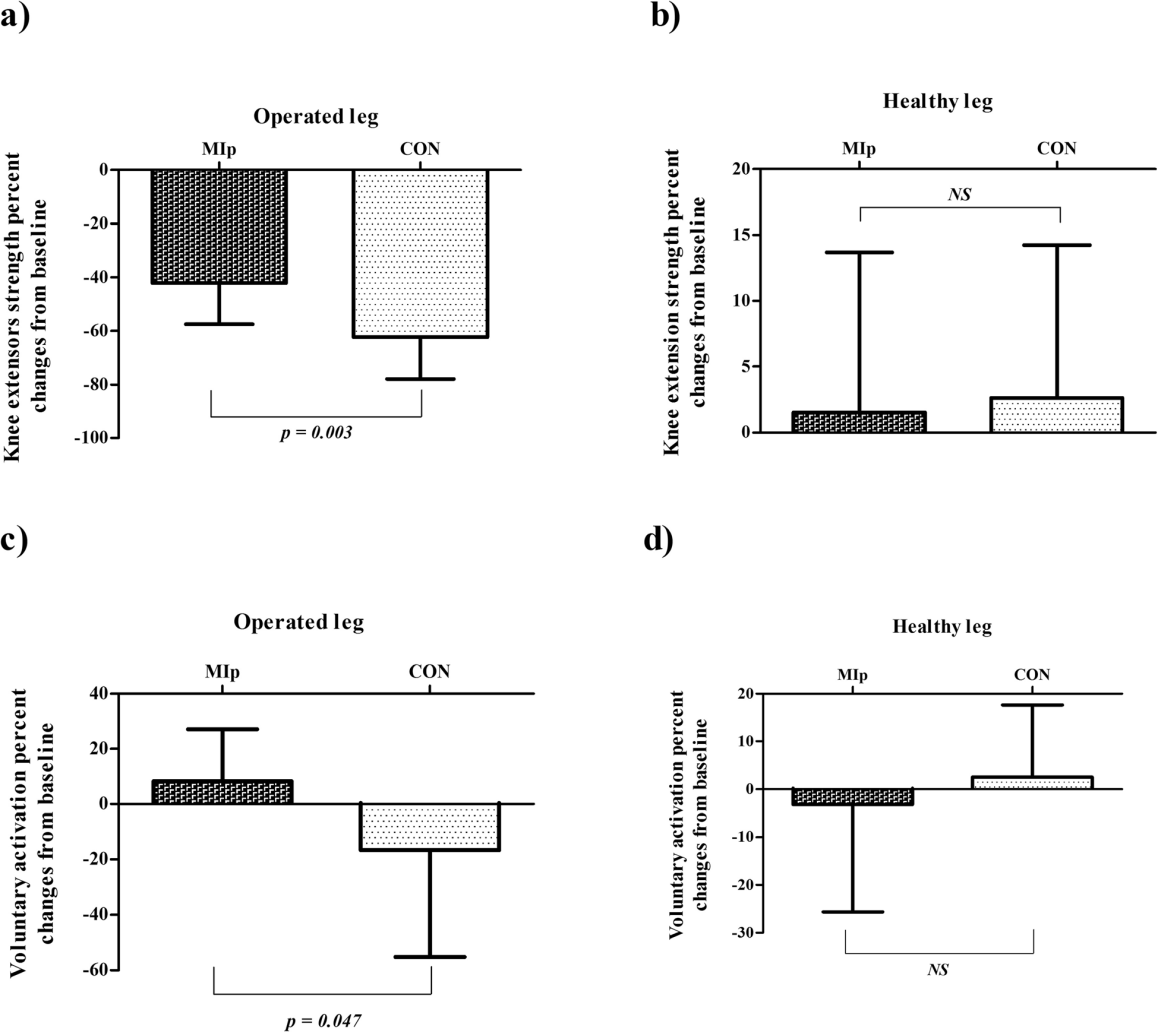
Mean percent changes (and standard deviations) from baseline in knee extensor muscles strength (**a**, **b**) and voluntary activation (**c**, **d**) in motor imagery practice (MIp) and control (CON) groups following total knee arthroplasty surgery

Both MIp and CON groups had significantly lower knee extensors strength of the operated leg at 1 month following surgery, when compared to PRE (*F*_1,24_ = 146.551, *p* < 0.001, *η*^*2*^ *=* 0.859). There was a significant time × group interaction effect (*F*_1,24_ = 9.215, *p* = 0.006, *η*^*2*^ *=* 0.277). Post hoc analysis showed that the MIp experienced a significantly lower decrease in strength compared to the CON group (− 42.32 ± 15.20% vs. − 62.46 ± 15.72%, *p* = 0.003) (Fig. 4).

Voluntary activation of knee extensor muscles was significantly lower in the CON group at post-surgery, when compared to PRE (− 16.66 ± 38.61, *p* = 0.036). A non-significant main effect was observed (*F*_1,24_ = 2.149, *p* = 0.156, *η*^*2*^ *=* 0.082), with significant time × group interaction effect (*F*_1,24_ = 7.042, *p* = 0.014, *η*^*2*^ *=* 0.227). Post hoc analysis showed that the MIp maintain their pre-operative levels of voluntary activation (8.28 ± 18.84%, *p* = 0.246), while significant differences were observed between groups at post-surgery (*p* = 0.047) (Fig. 4).

#### Non-operated leg

Both MIp and CON groups showed no significant changes in the strength of the non-operated leg when compared to PRE (both *p* ≤ 0.551). There were neither significant main (*p* = 0.551) nor interaction effects (*p* = 0.833) (Fig. 4).The same was found for voluntary muscle activation of the non-operated leg, which was not significantly altered post-surgery (both ≤ 0.423). There were neither significant main (*p* = 0.661) nor interaction effects (*p* = 0.341) (Fig. 4).

### Knee flexion and extension range of motion

#### Operated leg

There was a significant main effect for both flexion (*F*_1,24_ =10.352, *p* = 0.004, *η*^*2*^ *=* 0.301) and extension (*F*_1,24_ = 8.999, *p* = 0.006, *η*^*2*^ *=* 0.273). There were no significant time × group interaction effect for both flexion (*p* = 0.168) and extension (*p* = 0.824) of the operated leg at 1 month following surgery.

### Non-operated leg

Both MIp and CON groups showed no significant changes in flexion or extension of the non-operated leg when compared to PRE (both *p* ≤ 0.668). There were neither significant main (both *p* ≤ 0.417) nor interaction effects (*p* = 0.430).

### Timed Up-and-Go Test (TUG)

**Fig. 5:**
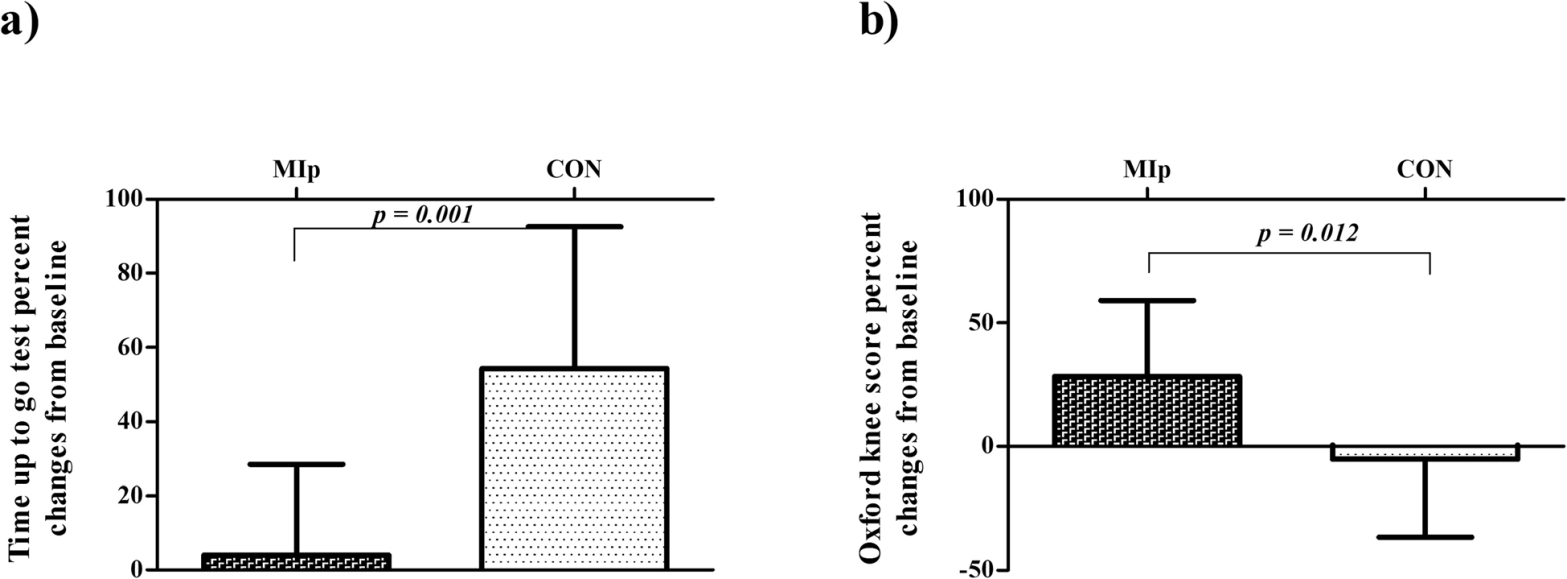
Mean percent changes (and standard deviations) from baseline in Timed Up-and-Go test (**a**), and Oxford Knee Score questionnaire (**b**) in motor imagery practice (MIp) and control (CON) groups following total knee arthroplasty surgery

There was a significant time × group interaction effect (*F*_1,24_ = 21.355, *p* < 0.001, *η*^*2*^ *=* 0.471). Post hoc analysis showed that the MIp maintained their level of performance between pre- and post-surgery (+ 4.02 ± 24.55%, *p* = 0.888), while there was a significant decrease in the CON group, displayed as more time taken for the TUG test at post-surgery (+ 54.38 ± 38.17%, *p* < 0.001). In addition, a significant difference between groups was observed post-surgery (*p* = 0.001) (Fig. 5).

### Knee pain

Both the MIp and CON groups showed significantly lower knee pain in the operated (MIp − 39.79 ± 21.19; CON − 27.29 ± 32.6; both *p* < 0.05) and non-operated leg (MIp − 83.33 ± 35.63; CON − 56.0 ± 49.71; both *p* < 0.05) at 1 month following surgery, when compared to PRE. However, there were no significant interaction effects (both *p* ≤ 0.455).

### Maximal grip strength

Both MIp and CON groups showed no significant changes in grip strength when compared to PRE (*p* = 0.456). There were neither significant main (*p* = 0.624) nor interaction effects (*p* = 0.416).

### Oxford Knee Score (OKS) questionnaire

There was significant time × group interaction effect (*F*_1,24_ = 9.254, *p* = 0.005, *η*^*2*^ *=* 0.280). Post hoc analysis showed that the MIp experienced significant increase in OKS score between pre- and post-surgery (+ 28.26 ± 30.86%, *p* = 0.018), with no significant deterioration in the CON group (− 5.16 ± 31.52%, *p* = 0.163). In addition, a significant difference between groups was observed post-surgery (*p* = 0.012) (Fig. 5).

### Regression analysis

**Fig. 6:**
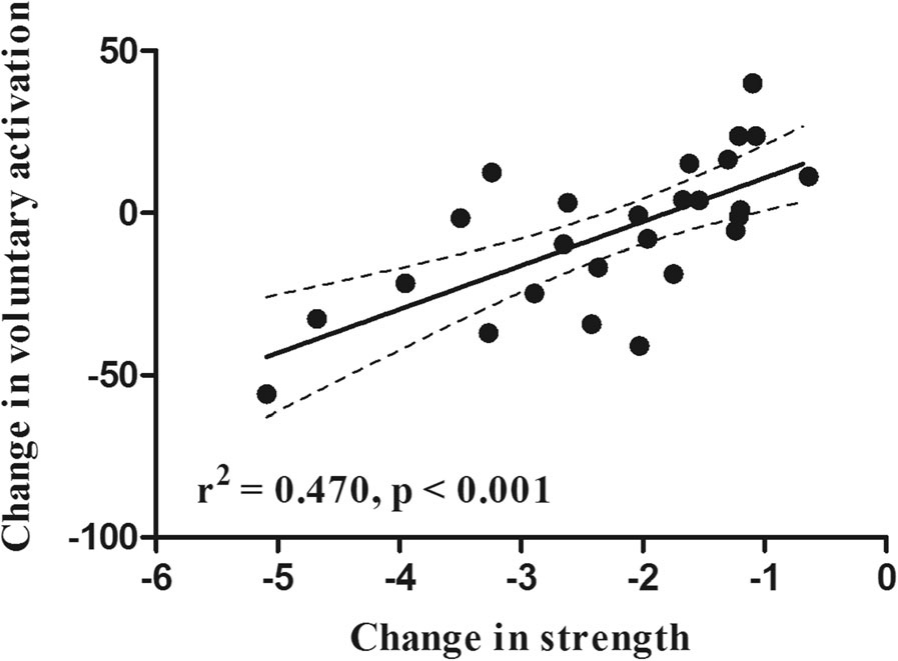
Linear regression analysis of the contribution of the change in voluntary activation to the change in the strength of the quadriceps muscle of the operated leg

Multiple regression analysis revealed that delta change in voluntary muscle activation and delta change in perceived pain explained 47.3% of the relative change in the operated leg quadriceps muscle strength (*r*^2^= 0.474, *p* = 0.001). However, voluntary muscle activation alone accounted for 47% of the observed strength loss of the quadriceps (*r*^2^ = 0.470, *p* < 0.001) (Fig. 6), while pain accounted for 2.6% only (*r*^2^ = 0.026, *p* = 0.428).

## Discussion

This study compared the short-term effects of MI practice in addition to routine physical therapy on VA and other objective and subjective measures related to motor performance in TKA patients. The major findings were that the patients in the MIp maintained pre-surgery levels of VA, experienced less deterioration of quadriceps muscle strength and functional mobility, and had significantly greater self-reported scores in physical function at POST when compared to controls. Further, there were no observed differences in knee range of motion or perceived level of pain. Also, MI practice intervention did not affect the motor performance of the non-operated leg in any of the variables assessed, suggesting that there were no contralateral limb effects. In addition, multiple linear regression analysis showed that the failure of VA explained 47% of the quadriceps muscle strength loss, with no significant contribution of the perceived level of pain.

Recovery of quadriceps muscle strength has recently received considerable attention, being a major determinant of general physical function following a TKA [6, 40]. Accordingly, in the early acute post-surgery period a variety of factors could alter the quadriceps muscle function, including joint damage [41, 42], pain [43], use of a tourniquet during operative procedure [44], inflammation [45], and post-operative knee swelling [8] eventually inducing arthrogenic muscle inhibition [46]. Quadriceps arthrogenic inhibition is commonly assessed by VA measurement and plays a significant role in quadriceps weakness, explaining approximately 60% of quadriceps muscle strength loss from pre-to-post surgery [7, 47]. Similar to previous studies [7, 48], voluntary activation explained nearly half of the quadriceps muscle strength loss. Hence, our hypothesis that MI practice can modify proxies of central factors of movement control was confirmed, demonstrating significant negative alterations of VA in controls only (− 16.7%), while MIp showed a non-significant increase of 8.3% on average. Although quadriceps strength of the operated leg was significantly altered following surgery, the MIp maintained greater pre-operative strength compared to the control, at 1 month post-surgery. This could be directly related to preserved muscle activation. This magnitude of difference in response is similar to that reported in previous studies (Table 5) [21, 49, 50]. For example, 26.9% and 19.9% of difference favouring high-intensity rehabilitation vs. low-intensity rehabilitation [49] and neuromuscular electrical stimulation (NmES) vs. RPT [50] were found in TKA patients, respectively. Moreover, Moukarzel et al. [21] investigated the effects of MI practice as an adjunct tool to combined NmES and RPT rehabilitation: the MI group gained 26.9% more strength than the control group [21]. The latter results support previous findings that combining the MI with NmES in addition to common physical therapy is beneficial to evoking re-learning of the motor task in stroke patients compared to NmES only [51, 52]. The provision of somatosensory inputs during MI influences motor performance improvement thanks to motor cortex plasticity [52], in a fashion similar to the actual execution of the movement [53]. While the methodological heterogeneity of the aforementioned studies is evident (i.e., in intervention applied to the experimental and/or control group and the time of baseline evaluation), our results showed comparable magnitude and direction of the effect of MI practice on the alleviation of strength loss in the early post-surgery period of TKA patients. Also, the central mechanisms of MI practice effectiveness were confirmed. Therefore, future studies should use a holistic approach and combine MI with NmES in addition to RPT.

**Table 5:** Comparison of published studies on operated leg quadriceps strength recovery in patients undergoing TKA before and 1 month after surgery

Study	Test mode	Time point	Types of intervention	Groups				
				PRE	POST	Percent of change	RDC (95% CI)	MBI
Bade and Stevens	Isometric 60° (Nm/kg)	3.5 weeks	HIRT	1.3 ± 0.5	1.0 ± 0.3	− 23.1	0.30 (− 0.11, 0.71)	Possibly beneficial
			LIRT	1.2 ± 0.4	0.6 ± 0.2	− 50.0		
Stevens-Lapsley et al	Isometric 60° (Nm/kg)	3.5 weeks	NmES	1.33 ± 0.57	0.93 ± 0.41	− 30.1	0.26 (0.02, 0.50)	Possibly beneficial
			Usual care	1.32 ± 0.49	0.66 ± 0.24	− 50.0		
Moukarzel et al.	Isometric HHD (Nm/BMI)	4 weeks	MIp	6.09 ± 0.55	13.30 ± 0.87	+ 118.4	1.59 (0.90, 2.28)	Most likely beneficial
			Usual care	6.14 ± 0.46	11.76 ± 0.82	+ 91.5		
Current study	Isometric 60° (Nm/kg)	4 weeks	MIp	1.37 ± 0.35	0.80 ± 0.34	− 42.32	0.39 (0.03, 0.75)	Likely beneficial
			Usual care	1.51 ± 0.38	0.55 ± 0.25	− 62.46		

*HHD* Hand-held dynamometer, *HIRT* High-intensity resistance training, *LIRT* Low-intensity resistance training, *MBI* Magnitude-based inference, *MIp* Motor imagery practice group, *NmES* Neuromuscular electrical stimulation, *RDC* Raw mean difference in change

MI exerts positive effects on strength improvements following musculoskeletal injuries [54] and/or surgeries [21]. During mental simulation of movement, similar neurophysiological processes are activated as during actual movement execution [55, 56]. Thus, without mechanical stimulus on the musculoskeletal system, the underlying mechanism of those changes might be of neural origin only [57, 58]. Indeed, MI practice induces cortical reorganization [59]. Further, it evokes movement-related cortical potentials, enhancing corticospinal excitability and muscular activity, which that consequently leads to increased muscle force output [13, 60–62]. To the best of our knowledge, only one previous study tested the hypothesis that MI training increases voluntary neural drive to task-oriented muscle action assessed by the twitch interpolation technique. Following 8 weeks of isometric strength training of the elbow flexor muscles, imagined isometric training or active control group, revealed significant pre- to post-strength improvement in all groups, without any significant alterations in VA level.

Compared to our study, previously reported non-significant changes in voluntary drive might be ascribed to differences in the studied population (healthy young vs. older adults) and higher prior study levels of VA in trained muscle (96.2 ± 0.5% vs. MIp 80.08 ± 13.28% and CON 80.86 ± 14.13%). It is possible that MI practice does not provide enough stimuli to induce significant changes in the neural drive in healthy young adults with already high values of VA. On the contrary, it exerts beneficial effects in an older symptomatic population with chronically impaired knee function.

Considering the range of motion and knee pain, there were no significant changes following MI practice, in contrast to the findings of a recent study investigating MI training in TKA patients rehabilitation [21]. While we were concentrated on strength only, Moukarzel et al. [21] designed MI practice with several MI exercises, focusing on three different objectives: i.e., (i) knee pain management, (ii) flexibility, and (iii) quadriceps strength [21]. The patients demonstrated only training-specific changes (i.e., alleviated pain, enhanced range of motion, and quadriceps strength), without effects that went beyond the trained task (e.g., functional mobility assessed by TUG test or knee swelling). The positive association of strength and TUG performance was confirmed previously [63]. Mizner et al. [63] found that functional performance assessed by TUG, a stair climbing test, a 6 min walk test and a sit-to-stand was significantly related to quadriceps strength of both legs. Similar to previous studies [49, 50], we showed a significant improvement of TUG performance in the intervention group compared to controls. Thus, contradictory findings might be explained by two major factors: (a) when compared to the study by Moukarzel et al. [21], in all the aforementioned studies, including the present one, the control groups used routine physical therapy (not high-intensity rehabilitation and/or NmES treatment) only, and this might lead to greater loss in performance [49, 50]; (b) different MI approaches used, with ours focusing strictly on strength domain, rather than other symptoms of TKA patients. That is also possibly why there were no significant differences in pain and range of motion measured in the current study.

Self-reported physical function assessed by questionnaires has been increasingly used to assess knee function following TKA surgery [38, 64]. In the current study, we used the OKS questionnaire, which is a suitable instrument to assess knee function both before and after TKA [38]. However, the OKS score is pain-dependent, suggesting that patients’ reporting of functional status is influenced more by their level of perceived pain rather than their ability to perform the task [65]. In the present study, the MIp showed significant improvement in OKS score when compared to controls, while there were no significant changes in pain level after surgery. Therefore, MI practice positively influences self-reported knee function, independent of the patients’ pain level.

Some limitations of the present investigation should be highlighted. First, patients were not provided with the fast track model of TKA perioperative practice, which is cost- and time-effective, showing benefits on patients’ function and satisfaction as well [66]. Second, our rigorous inclusion criteria narrowed the general conclusions of MI intervention efficiency in TKA patients (e.g., those with a BMI of 40 kg/m^2^ or higher and/or receiving bilateral TKA). Third, patients of three different surgeons were included in the current study. However, all three had over 10 years of experience and used the same surgical technique. Fourth, a 24% loss to follow up might be considered as a limitation of the current study; however, it reflects a real-life scenario which is presumed to happen in clinical studies assessing older patients after major surgery [67]. Also, to alleviate results of interpretation bias from patients lost to follow up, we conducted three-fold analysis, showing no discrepancies between observed results. Future studies should investigate the influence of MI practice on TKA patients with different comorbidities as well as those in other countries, as health care delivery systems and TKA perioperative protocols vary.

## Conclusion

In summary, to our knowledge, this is the first study analysing the effects of MI practice on voluntary activation of the quadriceps muscle and self-reported measure of physical function in patients who underwent TKA surgery. The addition of MI practice to routine physical therapy initiated within 48 h after TKA preserved the pre-operative level of voluntary activation of the quadriceps muscle and attenuated both objective and subjective measures of physical function at 1 month after TKA. However, the performance of the non-operated leg was not altered, suggesting that MI practice did not exert any statistically significant effect on the contralateral limb for the variables considered in this investigation. MI practice might be a suitable adjunct therapeutic tool to common rehabilitation practice for TKA patients in the early postoperative period.

## Data Availability

The data set of the current study is available as supplementary material.

## Abbreviations

MI: Motor imagery
VA: Voluntary activation
TKA: Total knee arthroplasty
MIp: Motor imagery practice
CON: Control group
PRE: Evaluation before surgery
POST: Evaluation after surgery
TUG: Timed up-and-go test
MViC: Maximal voluntary isometric strength
OKS: Oxford knee score
OA: Osteoarthritis
RPT: Routine physical therapy
